# Supplementary material for: MetaRibo-Seq measures translation in microbiomes
Source: Nat Commun. 2020 Jun 29;11:3268. doi: 10.1038/s41467-020-17081-z (PMC7324362; doi:10.1038/s41467-020-17081-z)
Supplement: Supplementary file 10 — Supplementary Data 7 [file 41467_2020_17081_MOESM10_ESM.zip › File2/Confidence_High_Taxonomy/345529_out.krona.html]

Javascript must be enabled to view this page.

members
magnitude
magnitudeUnassigned
count
unassigned
taxon
rank

345529\_out

113

2
superkingdom
113

phylum
113
976

113
class
200643

171549
113
order

815
112
family


SRS011134\_contig\_number\_52899SRS013476\_contig\_number\_41634SRS016139\_contig\_number\_contig-100\_3573.3574SRS017701\_contig\_number\_contig-100\_949.129162SRS017821\_contig\_number\_contig-100\_309.165738SRS022609\_contig\_number\_contig-100\_1207.261566SRS024132\_contig\_number\_8167SRS024331\_contig\_number\_contig-100\_1558.195010SRS046068\_contig\_number\_contig-100\_3259.44941SRS063040\_contig\_number\_50882SRS1041136\_contig\_number\_23744SRS146812\_contig\_number\_contig-100\_1220.311561SRS148091\_contig\_number\_2442SRS893292\_contig\_number\_contig-100\_519.66280SRS893300\_contig\_number\_contig-100\_742.80196
genus
816
15
112

species
3

SRS023715\_contig\_number\_14712SRS058770\_contig\_number\_15572SRS971276\_contig\_number\_28708
46506


SRS014613\_contig\_number\_2651SRS023346\_contig\_number\_4123
818
species
2

species
14

SRS017191\_contig\_number\_14931SRS020394\_contig\_number\_18426SRS022137\_contig\_number\_contig-100\_1345.125340SRS043701\_contig\_number\_contig-100\_606.115249SRS048060\_contig\_number\_13440SRS064232\_contig\_number\_contig-100\_250.42050SRS064276\_contig\_number\_29094SRS101433\_contig\_number\_contig-100\_1838.96873SRS1041145\_contig\_number\_29107SRS1054691\_contig\_number\_1900SRS1055067\_contig\_number\_contig-100\_1814.100413SRS1055099\_contig\_number\_11224SRS147653\_contig\_number\_6349SRS893342\_contig\_number\_10150
2292935

2292923

SRS013215\_contig\_number\_791SRS024549\_contig\_number\_5570
2
species

821

SRS013951\_contig\_number\_39044SRS024663\_contig\_number\_contig-100\_435.93979SRS043001\_contig\_number\_7089SRS045826\_contig\_number\_23064SRS058070\_contig\_number\_24901SRS063985\_contig\_number\_30166SRS075341\_contig\_number\_12048SRS1041038\_contig\_number\_16206SRS1055043\_contig\_number\_1236SRS143181\_contig\_number\_15301SRS143523\_contig\_number\_24904SRS144135\_contig\_number\_32341SRS148159\_contig\_number\_50149SRS148319\_contig\_number\_13570
14
species

species
1

SRS1041032\_contig\_number\_11008
329854

species
3

SRS013940\_contig\_number\_20842SRS055982\_contig\_number\_18381SRS077024\_contig\_number\_3875
28116

43
species
357276

SRS012273\_contig\_number\_contig-100\_781.265986SRS013098\_contig\_number\_48775SRS015264\_contig\_number\_21220SRS015794\_contig\_number\_12771SRS016018\_contig\_number\_10484SRS017103\_contig\_number\_42632SRS018049\_contig\_number\_739SRS018133\_contig\_number\_14191SRS018575\_contig\_number\_contig-100\_659.60126SRS019286\_contig\_number\_19SRS019685\_contig\_number\_contig-100\_2021.218029SRS019685\_contig\_number\_contig-100\_2109.218117SRS020869\_contig\_number\_38791SRS022071\_contig\_number\_4763SRS023583\_contig\_number\_18351SRS042628\_contig\_number\_34081SRS043768\_contig\_number\_30365SRS045713\_contig\_number\_contig-100\_529.114557SRS046717\_contig\_number\_7376SRS048870\_contig\_number\_22128SRS052078\_contig\_number\_490SRS054590\_contig\_number\_98SRS056519\_contig\_number\_2178SRS057717\_contig\_number\_1841SRS064757\_contig\_number\_22257SRS065504\_contig\_number\_contig-100\_445.215816SRS075021\_contig\_number\_6185SRS075878\_contig\_number\_contig-100\_852.147149SRS077086\_contig\_number\_contig-100\_153.70871SRS077127\_contig\_number\_6958SRS077392\_contig\_number\_43366SRS098717\_contig\_number\_13765SRS101268\_contig\_number\_72SRS1041039\_contig\_number\_27147SRS104636\_contig\_number\_13710SRS105153\_contig\_number\_37653SRS140645\_contig\_number\_13SRS142712\_contig\_number\_589SRS148511\_contig\_number\_4357SRS149784\_contig\_number\_19290SRS149879\_contig\_number\_24806SRS150029\_contig\_number\_20037SRS893230\_contig\_number\_11799


SRS022524\_contig\_number\_10426SRS045195\_contig\_number\_6369SRS048981\_contig\_number\_1428SRS050299\_contig\_number\_9742SRS098655\_contig\_number\_3630SRS1041112\_contig\_number\_3577SRS1055034\_contig\_number\_contig-100\_177.82904SRS140513\_contig\_number\_13085SRS148874\_contig\_number\_10848
817
species
9

2
species
371601

SRS016381\_contig\_number\_1033SRS018541\_contig\_number\_3343

species
1

SRS144183\_contig\_number\_30172
28119

1
species
820

SRS143598\_contig\_number\_9338

2
species
626931

SRS013800\_contig\_number\_27094SRS043667\_contig\_number\_contig-100\_186.35715

171550
family
1

239759
genus
1


SRS019161\_contig\_number\_2422
2292910
species
1
